# Improved production of polysaccharides in *Ganoderma lingzhi* mycelia by plasma mutagenesis and rapid screening of mutated strains through infrared spectroscopy

**DOI:** 10.1371/journal.pone.0204266

**Published:** 2018-09-21

**Authors:** Yuhan Ma, Qianqian Zhang, Qifu Zhang, Huaqi He, Zhu Chen, Yan Zhao, Da Wei, Mingguang Kong, Qing Huang

**Affiliations:** 1 Key Laboratory of High Magnetic Field and Ion Beam Physical Biology, Key Laboratory of Environmental Toxicology and Pollution Control Technology of Anhui Province, Institute of Technical Biology and Agriculture Engineering, Hefei Institutes of Physical Science, Chinese Academy of Sciences, Hefei, China; 2 University of Science & Technology of China, Hefei, China; 3 College of Life and Health Sciences, Anhui Science and Technology University, Fengyang, China; 4 Institute of Solid State Physics, Hefei Institutes of Physical Science, Chinese Academy of Sciences, Hefei, China; Helsingin Yliopisto, FINLAND

## Abstract

As a traditional Chinese medicine, *Ganoderma lingzhi* has attracted increasing attention for both scientific research and medical application. In this work, in order to improve the production of polysaccharides from an original wide-type (WT) strain (named "RWY-0") of *Ganoderma lingzhi*, we applied atmospheric-pressure dielectric barrier discharge (DBD) nonthermal plasma to the protoplasts of RWY-0 for mutagenesis treatment. Through a randomly amplified polymorphic DNA (RAPD) assay, at least 10 mutagenic strains were confirmed. They also showed different mycelium characteristics in terms of shape, color, size and biomass in liquid fermentation. The mutant strains were examined by infrared spectroscopy, and based on the established near-infrared (NIR) quantification model, the polysaccharide contents in these mutants were quantitatively evaluated. As a result, we found that the *Ganoderma* polysaccharide contents in some of the mutant strains were significantly changed compared with that of the original WT strain. The polysaccharide content of RWY-1 *G*. *lingzhi* was considerably higher than that of the WT strain, with an increase of 25.6%. Thus, this preliminary work demonstrates the extension of the plasma mutagenesis application in acquiring polysaccharide-enhanced *Ganoderma lingzhi* mutants and shows the usefulness of NIR spectroscopy in the rapid screening of mutagenic strains for other important ingredients.

## 1. Introduction

*Ganoderma lingzhi* is a basidiomycete white rot fungus that has been used as medicine in China for at least two thousand years [1]. Along with other Chinese herbal medicines, *G*. *lingzhi* was embodied in the American Herbal Pharmacopoeia and Therapeutic Compendium in 2000 [2], and since 2016, it has been adopted in the United States Pharmacopoeia as a medicinal raw material [3]. As a traditional Chinese herbal medicine, *G*. *lingzhi* has many pharmaceutical effects, such as immunomodulatory activity [4, 5], regulation of cardiovascular function [6, 7], regulation of blood glucose [8, 9], and a hepatoprotective effect [10, 11]. *Ganoderma* are particularly recommended for their immune-supporting effects [2], and the medicinal evidence comes from both animal experiments [11, 12] and clinical trials [13, 14]. *G*. *lingzhi* polysaccharides can enhance the host immunity and kill tumor cells when the host is implanted into tumor [15, 16], and *G*. *lingzhi* polysaccharides in particular can improve the immune ability of cancer patients treated with radiotherapy and chemotherapy [14].

To improve the production of pharmacological components of *Ganoderma lingzhi*, many workers have tried many different approaches in both research and application. One conventional approach is based on the method of cultivation, such as adjusting the components in culture medium, including a carbon source, nitrogen source, fed-batch, oxygen supplier, fungal elicitors, and hormone. For example, Zhao et al. showed that by limiting the glutamine content in the culture medium, the triterpene content of *Ganoderma lucidum* mycelium could be increased effectively [17]. Ren et al. showed that when the *Ganoderma* mycelium pellets were treated with methyl jasmonate during liquid fermentation, the synthesis of ganoderic acid in the mycelia was promoted [18]. Papinutti et al. studied the effect of maltose and glucose on the content of *Ganoderma lucidum* exopolysaccharides [19]. Wei et al. have shown that sucrose can significantly increase the yield of biomass, polysaccharide and ganoderic acid using the mixture of glucose and sucrose as a carbon source [20]. Tang et al. have studied the effects of dissolved oxygen, pH-shift and fed-batch fermentation on the production of *Ganoderma* polysaccharides [21–23]. Zhu et al. have reported the use of fungal elicitors to increase *Ganoderma* mycelia polysaccharide content [24]. Another approach is based on contemporary genetic engineering, such as the introduction of exogenous genes into *Ganoderma lingzhi* by genetic transduction. For example, Huan-Jun et al. transformed the Vitreoscilla hemoglobin (VHb) gene into *Ganoderma lucidum*, which improved *Ganoderma* polysaccharide production in submerged fermentation [25]. Xu et al. reported that overexpressing the alpha phosphoglucomutase gene could increase the yield of *Ganoderma* polysaccharides [26]. Ji et al. found that overexpression of the UDP glucose pyrophosphorylase (UGP) gene would increase the production of polysaccharides [27].

Although genetic engineering is an important approach to obtaining mutated microbial species, in many cases this mutagenesis approach also has limitations if the information about the target gene is not clear or if the genetic regulations are too complicated. From a commercial point of view, an alternative approach is based on random mutagenesis. This may have a special advantage in that the selected mutated strains (after appropriate mutant screening and breeding) are classified as nontransgenic modified strains so that they do not require genetically modified organism certification. This may be critical in the countries where products marked "nongenetically modified" are more readily accepted by the public [28]. For the random mutagenesis method, the common mutagens include chemical mutagens, such as azide compounds [29] and alkylating agents [30], as well as physical mutagens such as ionizing-radiation, including heavy particle-beams [31]. In fact, the random mutagenesis approach has also been applied in the mutation breeding of *Ganoderma lucidum*. For example, Peng et al. reported that the use of lithium chloride (a chemical mutagen) to treat *Ganoderma lucidum* protoplasts led to the highest yields of intracellular polysaccharides and triterpenoids, which were 37.50 and 40.81 mg/g, respectively. These were 568.45% and 373.43% higher than those of the original strain, respectively [32]. For the physical mutagens, a conventional way is to utilize ionizing radiation or heavy-particle-beams. However, both electromagnetic ionizing radiation (e.g., X-ray and gamma-rays) and ion-beams may cause potential permanent damage to the user and so they require additional safety control measures. In addition, the supply of such ionizing radiation usually requires highly expense, professional training, and additional safety protection.

In this regard, therefore, nonthermal plasma mutagenesis shows special advantages for it may overcome the abovementioned shortcomings, and in fact, it has currently gained increasing attention for its usage in mutagenesis [33]. In nonthermal plasma, in addition to the critical effects of charged particles and the induced reactive species [34], other factors, such as UV [35, 36] and ozone [37, 38], may also play important roles in the induction of gene mutations and the improvement of mutagenesis efficiency. In particular, as one of the popular plasma techniques, dielectric barrier discharge (DBD) nonthermal plasma has emerged to be an effective approach for microbe mutagenesis because it does not require a vacuum system and is characterized by a low temperature treatment, high concentration of active species, good uniformity of discharge, simple operation, rapid mutation, high mutation rate and strong controllability [39]. For example, Zhao et al. utilized plasma discharge to mutate a polyunsaturated fatty acids producing strain of *Schizochytrium sp* [40]. Liu et al. obtained a *Yarrowia lipolytica* M53 strain with high glycosyl alcohol content by plasma discharge mutagenesis [41]. Li et al. showed that after *Aspergillus terreus* was treated with ARTP mutagenesis, it reached a higher itaconic acid yield of 19.3 g/L with 36.01% sugar conversion [42]. Cao et al. obtained five desired oleic acid producing strains of the oleaginous microalgae *Chlorella pyrenoidosa* screened from a mutant library by plasma discharge [43].

In this work, therefore, we attempted to make use of DBD nonthermal plasma mutagenesis to not only enrich the germplasm resources of *G*. *lingzhi* but also, in particular, to improve the production of polysaccharides in *G*. *lingzhi* through plasma treatment. For this purpose, we used the polysaccharide-high-yield *G*. *lingzhi* strain named RWY-0 by us, which we discovered and preserved. In the research, we also employed a homemade, convenient DBD device and used this device to treat the protoplasts of RWY-0 to acquire a variety of mutated strains. With the aid of the randomly amplified polymorphic DNA (RAPD) assay, we then employed infrared (IR) spectroscopy to identify and examine the polysaccharide content in the mycelia of the mutated strains after liquid fermentation. To assess the high-yield polysaccharide strains quickly and conveniently, we employed near-infrared (NIR) spectroscopy for the inspection of the mutated strains and conducted quantitative evaluation based on our formerly established NIR quantification model. As a result, we have not only obtained the desired mutant strain with a higher production of polysaccharides but also shown the effectiveness of the application of NIR spectroscopy in the screening of mutants.

## 2. Materials and methods

### 2.1 Materials

Lywallzyme was purchased from the Guangdong culture collection center. Driselase was purchased from Sigma-Aldrich, Inc. Mannitol, anthracone, and agarose, analytically pure, were purchased from Sangon Biotech (Shanghai) Co., Ltd. Alternative random primers were synthesized by Sangon Biotech (Shanghai) Co., Ltd. Sulfuric acid (analytically pure) and potassium bromide (spectroscopically pure) were bought from the Sinopharm Chemical Reagent Co., Ltd. The DNAsecure Plant Kit (DP320) was purchased from Tiangen Biotech (Beijing) Co., Ltd. A phosphoglucomutase (PGM) Elisa Kit and glucose phosphate isomerase (GPI) Elisa Kit were purchased from Fu Life Industry Co., Ltd. (Shanghai).

### 2.2 *G*. *lingzhi* strains and culture condition

The *G*. *lingzhi* strain CGMCC 5.0026 was purchased from China General Microbiological Culture Collection Center (CGMCC) and the wild-type strain of RWY-0 was donated from the Anhui Science and Technology University. The *Ganoderma lingzhi* strains were incubated in liquid cultures. The mycelium pellets were grown in 250 mL flasks containing 150 mL of a Potato Dextrose Broth (PDB) culture and placed on a rotary shaker incubator at 150 rpm at 28°C for 14 days before collection for experiments.

### 2.3 Plasma mutagenesis

**Fig 1:**
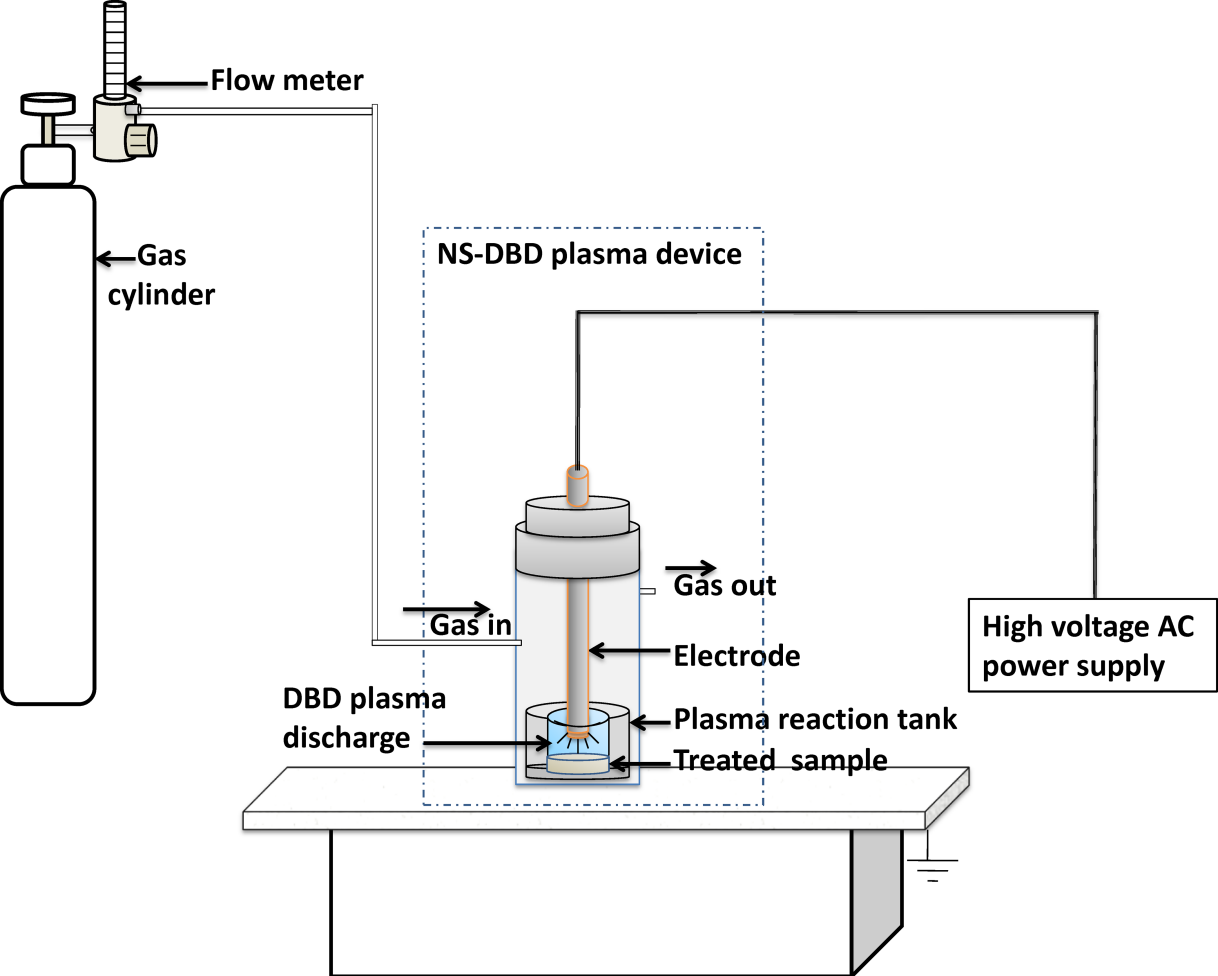
Schematic diagram of DBD plasma mutagenesis set-up.

The experimental instruments mainly include a UV-2550 UV-Vis spectrophotometer (Shimadzu Co., Ltd., Japan), FT-NIR spectrometer (MPA, Bruker Optik GmbH, Germany), Tissuelyser-24 automatic sample grinder (Shanghai Jingxin Industrial Development Co., Ltd, China), Applied Biosystems™ 2720 Thermal Cycler (ThermoFisher Scientific Co., Ltd, Singapore), Peiqing JS-2012 Gel imaging analysis system (Shanhai Peiqing Science & Technology Co., Ltd, China), and FD-1A-50 Lab Lyophilizer (Shanghai Bilon Instrument Co., Ltd., China). The dielectric barrier discharge (DBD) plasma mutagenesis device was homemade, and the experimental setup was built as shown in Fig 1. In the experiment, the nonthermal DBD plasma was working at atmospheric pressure and room temperature.

Before the plasma mutagenesis treatment, the preparation procedure of *G*. *lingzhi* liquid-cultured mycelia was as follows: under aseptic and sterile conditions, the liquid seed of *G*. *lingzhi* was inoculated in sterilized PDB liquid medium at 28°C and then shaken at 180 rpm for 14 days. The mycelium was taken out, centrifuged and washed with ddH_2_O on a clean bench. The *Ganoderma* mycelia, with an appropriate amount of 0.6 mol/L mannitol, were placed into the tissue homogenizer and ground on a clean bench. The precipitate was obtained by centrifugation at 3000 rpm for 5 min. The hyphal homogenate was treated by the compound enzymolysis method. The mycelia homogenate was added to a mixture of driselase and lywallzyme (at a final enzyme concentration of 20 mg/ml) and hydrolyzed at 30–35°C for 1.5 h. The lysate was filtered with sterile lens paper to remove nonhydrolyzed hyphae. The filtered liquid was collected and centrifuged at 1500 rpm for 3 min, and then the supernatant was discarded, and the precipitate (protoplasts) was retained. The protoplasts were checked with an optical microscope and counted with a blood cell plate, then dissolved and diluted with 0.6 mol/L mannitol to a concentration of 1×10^6^−10^7^ cells/ml. 200–500 μl of protoplast solution was pipetted into a quartz plasma reaction tank (φ = 2 cm) under sterile conditions. The quartz plasma reaction tank was gently shaken to make the protoplast solution evenly coat the bottom of the reaction tank.

For the plasma mutagenesis treatment, the plasma reaction electrode was placed on the surface of the protoplast solution for mutagenesis. The plasma reactor was sealed, and the working gas (either argon or helium) was passed into the reaction system for 4–6 minutes with a gas flow rate 1–2 L/min. The protoplasts were mutated at an operating voltage of 10–15 kV for 3–5 minutes. The DBD plasma treated protoplasts were collected, and 20 μl was pipetted onto the MYG regeneration medium. The DBD plasma treated solution was gently smeared onto the MYG solid medium. The protoplast smeared petri dishes were sealed and incubated in a 28°C for 3–5 days. After 3 to 5 days of regeneration, the protoplasts grew into microcolonies on the plate, and the microcolonies that grew faster and thicker were selected. The mycelia were picked out with sterilized toothpicks and inoculated on a petri dish covered with PDA solid medium. The DBD plasma treated strains were inoculated in the PDB liquid medium, PDA solid medium and preserved as seed strains at 4°C. For the liquid fermentation, the active components of the mycelia were determined after 14 days. The mutagenic effect was detected by the RAPD method, recorded and compared with the original strains.

### 2.4 Liquid fermentation of *G*. *lingzhi* mycelia

The DBD plasma-treated strains were inoculated in PDB liquid fermentation medium. After 3 generations of culture for 14 days, the mycelium of *G*. *lingzhi* was collected and washed with distilled water 3 times. The mycelium pellets were collected, photographed, freeze-dried in the lyophilizer, and the dry weights were recorded. The samples were ground into powder by an automatic sample grinder and then filtered with a sieve mesh 100 for subsequent measurements, including RAPD, intracellular polysaccharide (IPS), extracellular polysaccharides (EPS), phosphoglucomutase (PGM) and phosphoglucose isomerase (PGI) activity and the spectroscopy tests.

### 2.5 Detection of mutated *Ganoderma* strains by RAPD method

After further freezing with liquid nitrogen, 20 mg of lyophilized *Ganoderma* mycelia powder was ground with a sample grinder (60 HZ) for 5 min, and the genomic DNA was extracted using a Tiangen DNAsecure Plant Kit (DP320).

The alternative random primers are shown in Table 1. The D18 and D20 primers were suitable for this experiment throughout the preliminary experiments. The reagent composition included 1 μL *Ganoderma* genomic DNA templates (25 ng/μL), 1 μL random primers (10 μmol/L), 10.5 μL ddH_2_O, and 12.5 μL Taq enzyme (Nova Taq plus PCR Rorest Mix 2×).

**Table 1:** RAPD random primers for screening mutated strains.

Primer no.	Primers sequence
C5	GATGACCGCC
C6	GAACGGACTC
D18	GAGAGCCAAC
D20	ACCCGGTCAC
S24	AATCGGGCTG
S28	GTGACGTAGG
S42	GGACCCAACC
S47	TTGGCACGGG

For the PCR experiment, the samples were first denatured at 95°C, with 2 min on the initial cycle and 1 min on rest cycles. The samples were then annealed for 40 cycles at 37°C for 1 min, with an extension for 40 cycles at 72°C for 1 min, and a final 10 min extension. After a 1% agarose gel with EB was solidified at room temperature, 7 μL of the PCR products was added into the wells, and the samples were screened after 40 min of electrophoresis with a constant voltage of 110 V. For the protocols of polysaccharide measurement, the measurements of mid-IR spectra and NIR, refer to the our previous article [44].

### 2.6 Measurements of intracellular polysaccharide (IPS) and extracellular polysaccharides (EPS)

For the IPS and EPS measurements, the culture media were centrifuged at 3500 rpm for 5 min. The precipitate was used for IPS inspection [44], while the supernatant was used for EPS inspection. Anhydrous ethanol was added to the supernatant to a final concentration of 80%, cooled at 4°C overnight and then centrifuged at 10000 rpm for 5 min to obtain a precipitate. The content of polysaccharides was determined with the anthrone-sulfate method [44].

### 2.7 Evaluation of phosphoglucomutase (PGM) and phosphoglucose isomerase (PGI) activities

The PGM and PGI activities were evaluated using a phosphoglucomutase (PGM) Elisa Kit and a glucose phosphate isomerase (GPI) Elisa Kit. The main operation steps are as follows: 40 μL of liquid *G*. *lingzhi* mycelia or standard samples were added to an enzyme standard coating plate with the reaction reagent, sealed and kept at 37°C for 30 minutes. After washing the plate 5 times, the enzyme labeled reagent was added and the reaction took place at 37°C for 30 min. After washing the plate 5 times, the chromogenic agents A and B were added and then kept for 10 minutes at 37°C. The absorbance value was measured within 15 minutes after adding the terminating agent, and the enzyme activity was evaluated according to the standard curves.

### 2.8 Analysis of polysaccharide in *Ganoderma* mutant strains by infrared spectroscopy measurements of mid-IR spectra

Two milligrams of dried sample was added to 0.15 g potassium bromide crystal, ground into fine powder, pressed into a 13 mm tablet, and then placed into a Bruker ALPHA-T instrument (Bruker Optics GmbH, Ettlingen, Germany) for detection. The spectra were detected with a resolution of 4 cm^-1^ and 64 scans per sample [44].

#### Measurement of NIR spectra

Dried powder samples (0.3 g) were placed into a quartz detection cuvette and then into a FT-NIR spectrometer (NIR MPA, Bruker Optik GmbH, Germany) for detection. Each sample was tested several times and then averaged [44].

#### Data analysis

Both NIR and mid-IR spectral data were analyzed by using OPUS software (Bruker Optik GmbH, Ettlingen, Germany). (Bruker Optik GmbH, Ettlingen, Germany). Before further testing, all the spectra were pretreated using the procedures of vector normalization and baseline correction. For evaluation of the polysaccharide contents using the NIR quantitative model, which was established by our previous work [44], we input all the newly measured NIR spectra into the worksheet provided by the OPUS software, and after a calculation by the computer based on the NIR quantification model, the predicted values were then produced.

## 3. Results and discussion

### 3.1 Confirmation of mutagenized *G*. *lingzhi* strains

**Fig 2:**
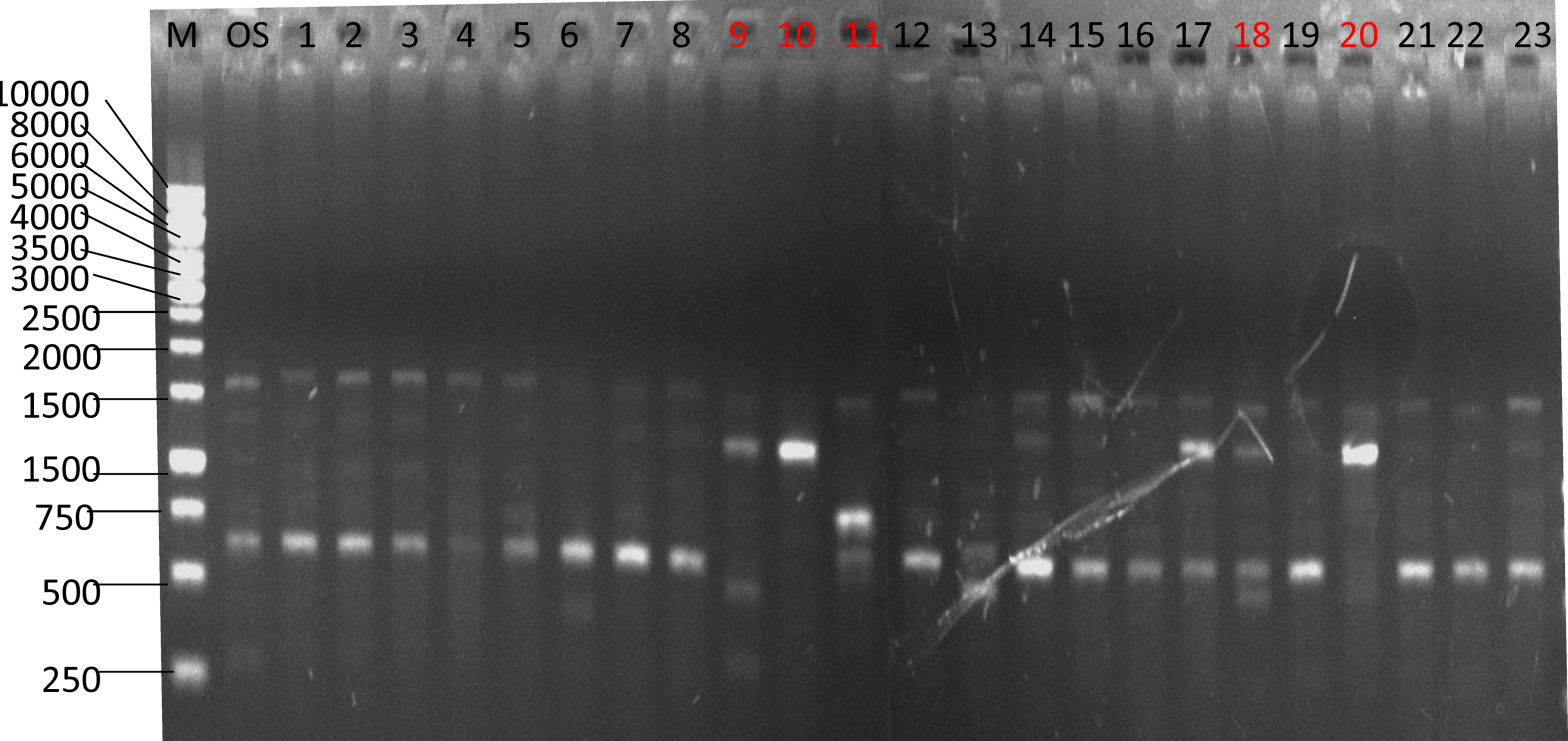
RAPD electrophoresis test for mutated *Ganoderma* strains.

The RAPD assay is a common and useful method for the identification of fungal strains and for the identification of *Ganoderma lucidum* strains [45, 46]. It is often used in mutagenesis identification as well [34, 47, 48]. In this work, we used RAPD to screen and identify the DBD plasma mutagenized *G*. *lingzhi* strains. The RAPD random primers are listed in Table 1. As a result, we have confirmed at least 10 mutagenized strains, which are marked as RWY-N (N = 1–10). A typical RAPD electrophoresis result is shown in Fig 2, with other evidence given in the Supporting Information (S1 Fig). The RAPD assay clearly shows the inter- and intra-specific genetic diversity in the plasma-mutagenized strains of *Ganoderma lingzhi*.

**Fig 3:**
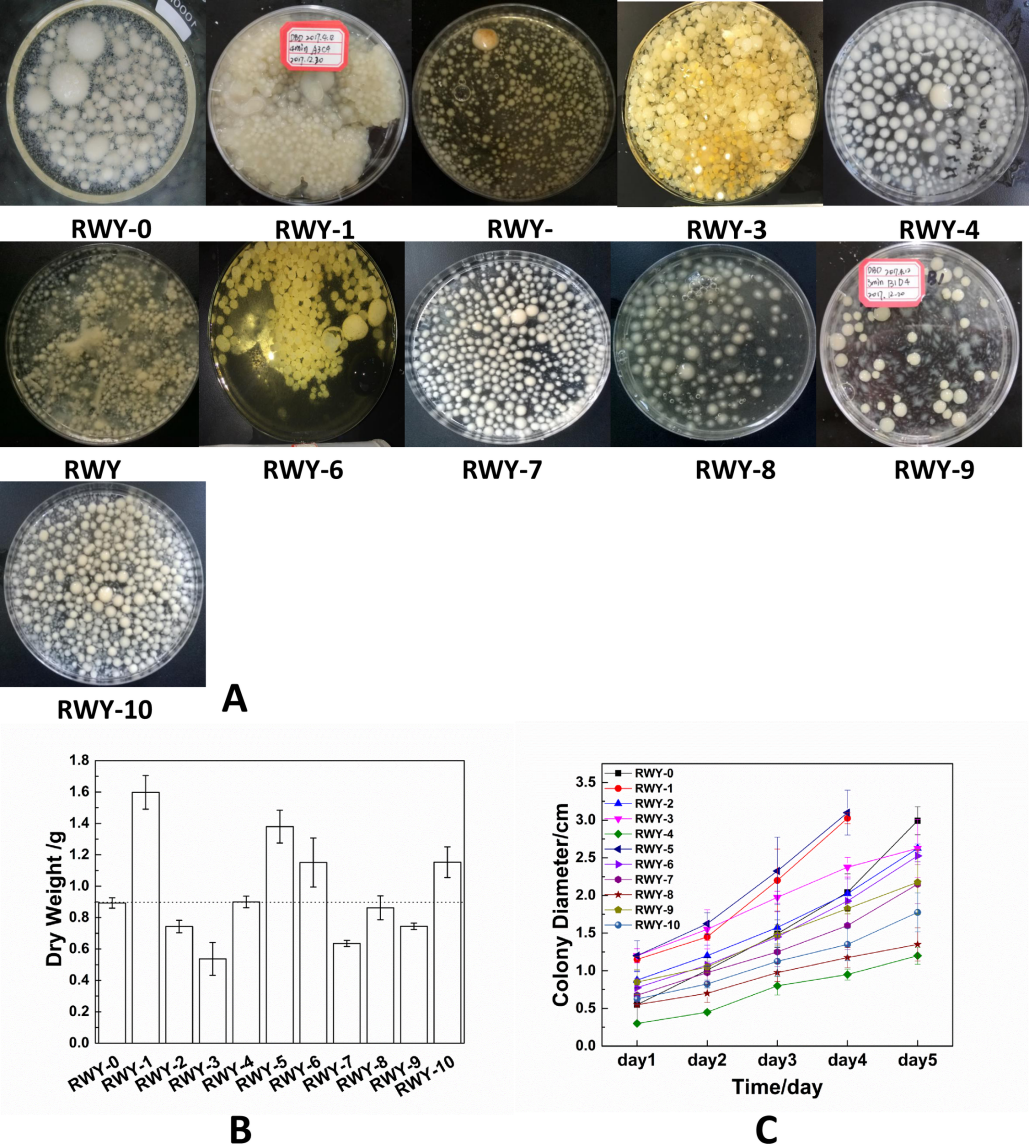
The DBD-plasma-induced mutated *G*. *lingzhi* strains. A: photographs of mutated mycelium pellets; B: dry weight of liquid fermentation; C: growth rate in solid culture media.

Furthermore, the phenotypes of the mutated *G*. *lingzhi* mycelia in liquid culture were also compared to distinguish the strains. After inoculation in PDB liquid medium for 14 days, the growth of the mutated *Ganoderma* strains was observed and recorded. The mutated mycelium pellets are shown in Fig 3(A). As seen in the photographs, all the mutated mycelium pellets show obvious differences in mycelium morphology, growth rate and surface color. The mutated strains RWY-5 and RWY-1 showed higher dry weights and growth rates, as illustrated in Fig 3(B) and 3(C).

### 3.2 Assessment of *Ganoderma* polysaccharides in mutated strains by infrared spectroscopy

As one of most important antitumor compounds in *G*. *lingzhi*, the *Ganoderma* polysaccharides have many pharmacological functions, such as promoting the proliferation of macrophages [49], activating lymphocytes [5] and regulating the production of cytokines [50] in vivo. Therefore, the content of *Ganoderma* polysaccharides in *G*. *lingzhi* is normally used as a representative evaluating indicator for the judgment of quality of the *Ganoderma* strains [51]. Therefore, in this work we were concerned with *Ganoderma* polysaccharides and employed infrared spectroscopy to assess the *Ganoderma* polysaccharides in the mutated strains. Infrared spectroscopy can probe the vibrations of the chemical groups in molecules, and therefore, it can also be used to certify the changes in biological composition and contents [52]. In fact, our previous work demonstrated that infrared spectroscopy is an effective, rapid and nondestructive method for the determination of *Ganoderma* polysaccharide levels [44].

**Fig 4:**
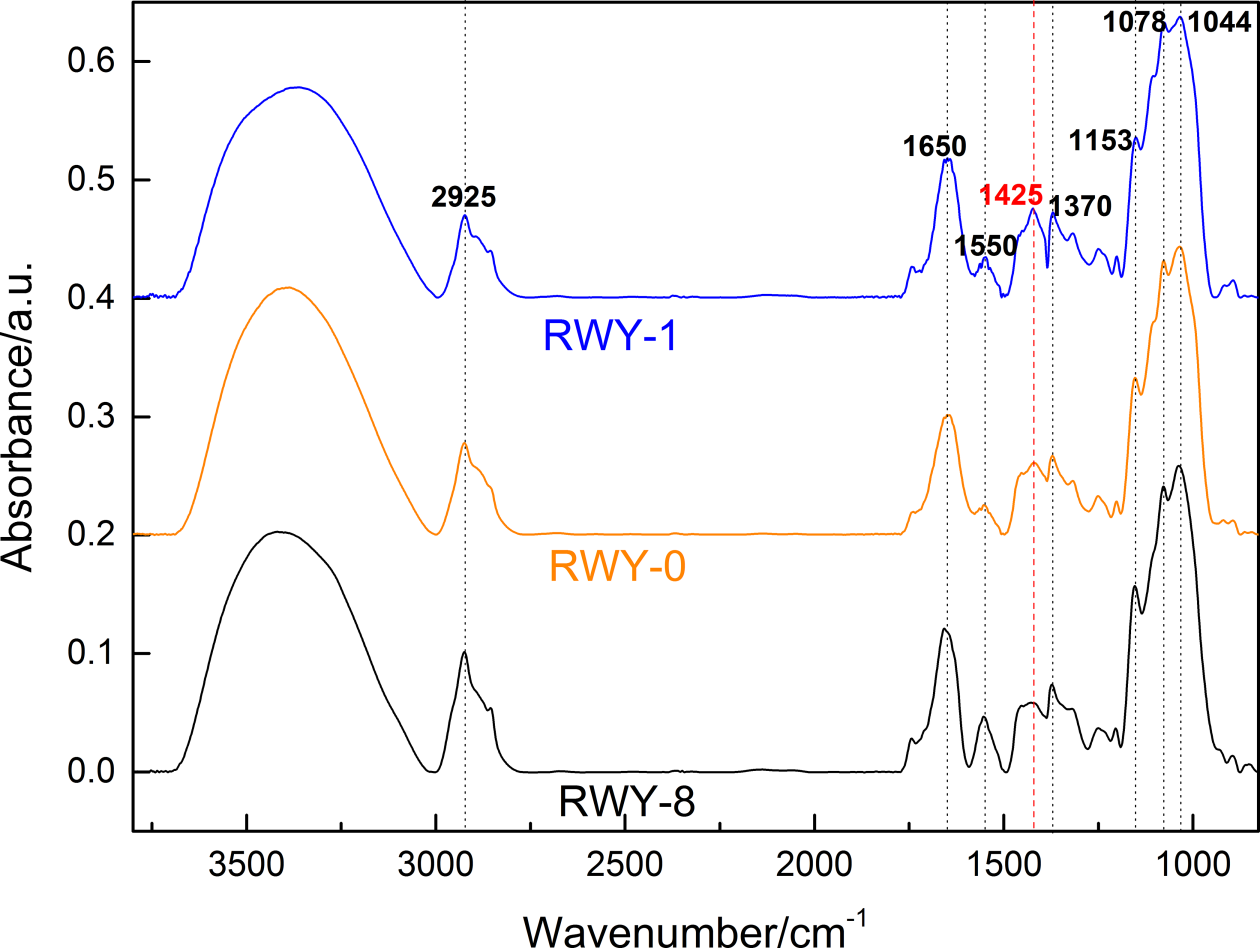
The mid-IR spectra of *G*. *lingzhi* strains (RWY-0, 1, 8).

For the qualitative assessment, we first employed mid-infrared (mid-IR) spectroscopy to examine the mutagenized *G*. *lingzhi* strains. Previous work has shown that mid-IR spectroscopy can be effectively utilized to identify the mutants. For example, Liu and Huang reported that mid-IR and Raman spectroscopy can be used for mutated *Haematococcus pluvialis* detection and screening [53]. Cote et al. reported that the changes of polysaccharide structure in *Leuconostoc mesenteroides* mutant strains can be detected with mid-IR spectra [54]. Galichet et al. showed that a change in the cell wall component of *Saccharomyces cerevisiae* after genetic transformation caused a change of mid-IR spectrum, so the mutagenized strain could be detected and screened by mid-IR spectroscopy [53, 55]. Our previous studies have also proven that the polysaccharide content in *G*. *lingzhi* mycelia can be judged qualitatively by mid-IR spectroscopy, especially according to an analysis of the characteristic peaks at 1425 and 1078 cm^-1^ [44]. Therefore, in this study, the dried mycelia of mutated strains were also qualitatively analyzed by mid-IR spectroscopy. In fact, the typical mid-IR spectra of mutagenized *G*. *lingzhi* strains are shown in Fig 4, with the assignments given in S1 Table. In particular in the mid-IR spectra, RWY-1 (higher-yield of polysaccharides) shows the strongest absorption peak at 1425 cm^-1^, while RWY-8 (lower-yield polysaccharides content) shows the weakest absorption peak at 1425 cm^-1^ in comparison with that of the original WT strain RWY-0.

**Fig 5:**
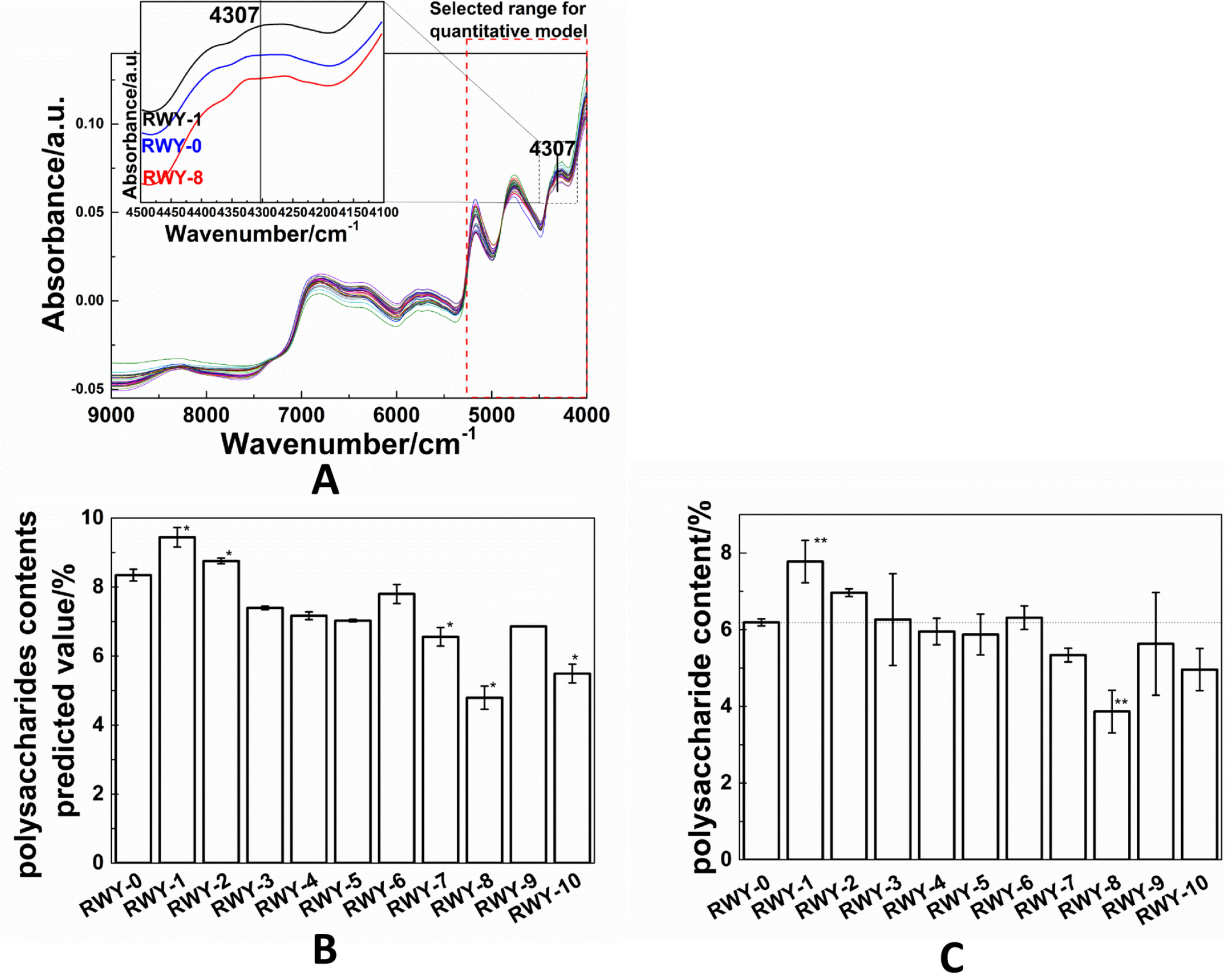
Evaluation of the polysaccharide contents of *Ganoderma* mutants. A: NIR spectra of mutated *Ganoderma* mycelia; B: the predicted polysaccharides contents of *Ganoderma* mutants based on spectra A; C: the measurement of polysaccharide contents of *G*. *lingzhi* mutants based on the anthrone-sulfuric acid method for comparison.

Furthermore, we employed near-infrared (NIR) spectroscopy for inspection of the mutants. NIR spectroscopy has been used in the agriculture field for a long time [56] and it is characterized by the overtones and combinations of the corresponding molecular fundamental vibrations in the mid-IR region [57]. NIR spectroscopy can be used for quantitative analysis because it is less affected by moisture [58]. Previous studies have shown that NIR spectroscopy can be effectively utilized for analyzing algae and plant mutants, including plasma mutated *Haematococcus pluvialis* strains [59], space mutated tomatoes [60], and chemically mutated peanuts [61]. The NIR spectra of *G*. *lingzhi*, shown in Fig 5(A), display the obvious differences, especially in the region of 5268–4000 cm^-1^. Additionally, Fig 5(A) shows clearly that the high-polysaccharide-yield strain RWY-1 has a stronger peak at 4307 cm^-1^ than the WT (RWY-0) strain, while the low-polysaccharide-yield strain RWY-8 has a weaker peak at this position than the WT strain. The variation in NIR spectra is basically due to the chemical component changes in *Ganoderma* strains [44]. As explained in our previous paper [44], the 4307 cm^-1^ band corresponds to the C-H deformation which has the counterpart at 1425 cm^-1^ in its mid-IR spectrum. Therefore, this result is also consistent with our mid-IR analysis as discussed above.

For the quantitative evaluation of polysaccharide content in the mycelia of *G*. *lingzhi* strains, we then utilized the NIR quantification model which had already been established in our previous work [44]. The NIR data analysis was based on the first derivative of the NIR spectra (S2 Fig). The evaluation is illustrated by Fig 5(B) (see the values listed in S2 Table). From the NIR assessment, we can clearly distinguish that the polysaccharide contents of strains RWY-1 and RWY-2 are considerably higher than the polysaccharide content of the original WT strain. The highest content was found in the strain RWY-1 and the polysaccharide content is approximately 25.6% higher than that of the original WT strain.

**Fig 6:**
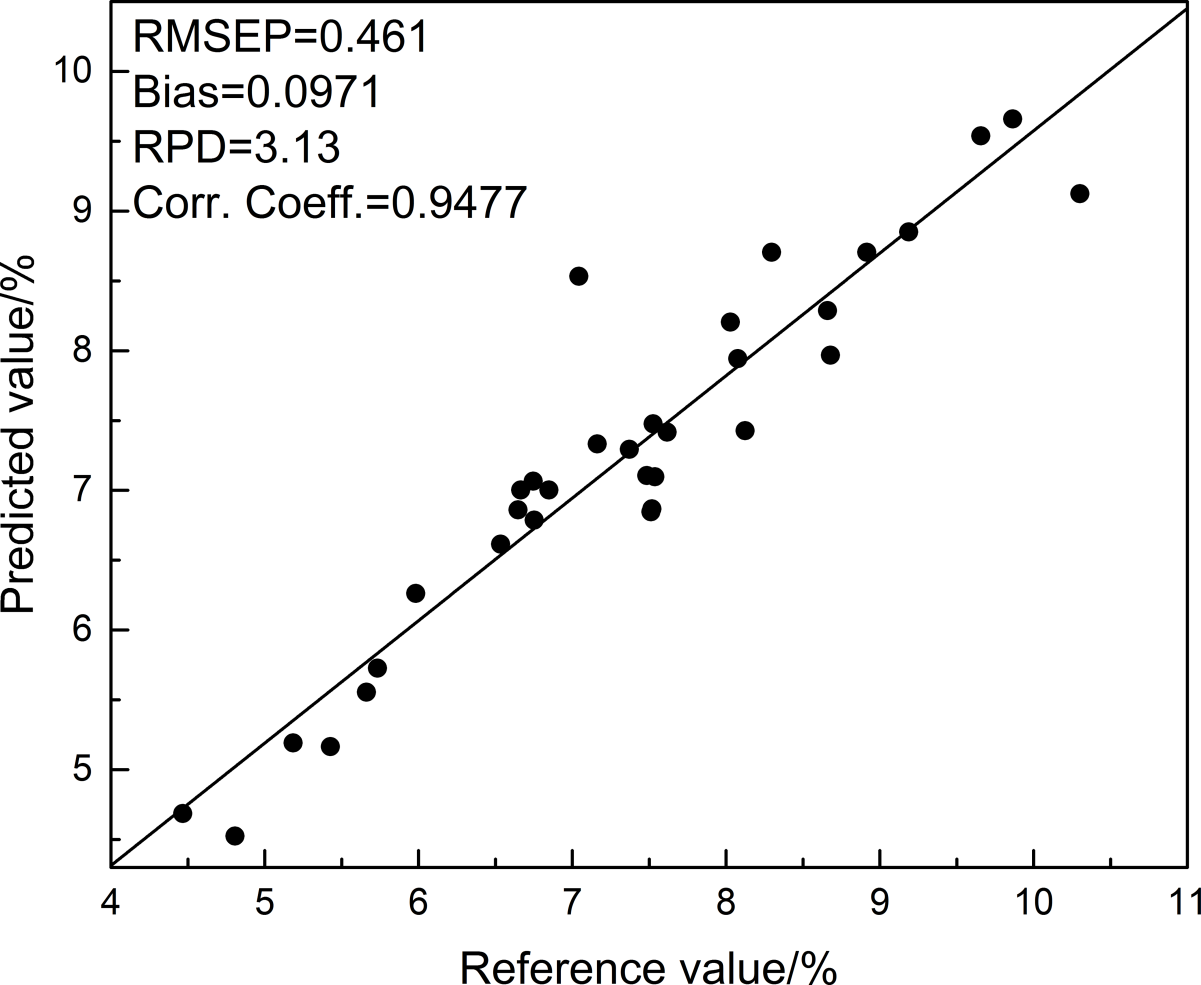
The correlation chart for NIR predicted and chemical values of polysaccharides in mutated *Ganoderma* strains.

Finally, we also checked the accuracy of the NIR quantitative assessment based on the conventional sulfuric acid anthrone method as used previously [44]. The results given in Fig 5(C) (see the values listed in S3 Table) for comparison validate the NIR assessment unambiguously. The correlation chart for the NIR predicted and chemical values is demonstrated in Fig 6, with the root mean square error of prediction (RMSEP) = 0.461, Bias = 0.0971, relative percent deviation (RPD) = 3.13, correlation coefficient (corr. coeff.) = 0.9477. The predicted values are in accordance with the chemical values, confirming the accuracy of the NIR quantification model for the assessment of *Ganoderma* polysaccharides in the mutated strains. To be noted, our NIR quantification model is valid not only for *Ganoderma lingzhi* from different origins but also for the strains mutated by plasma mutagenesis obtained in this work (S3 Fig).

### 3.3 Assessment of EPS content, PGM and PGI activities of G. lingzhi mutated strains

**Fig 7:**
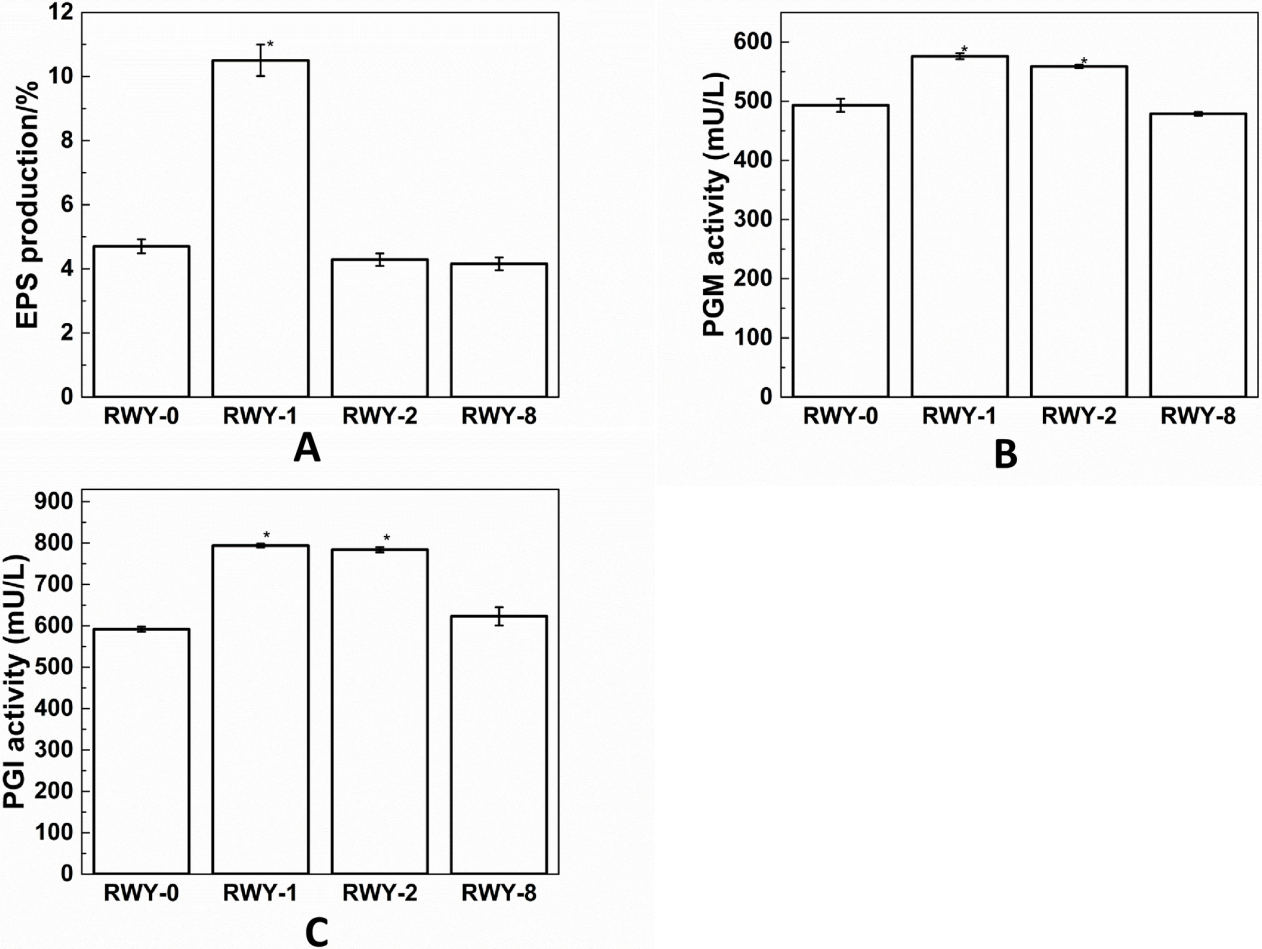
**The EPS content (A), PGM (B) and PGI activities (C) evaluated for the RWY-1, RWY-2, RWY-8, and RWY-0 mutated strains**.

To further verify the differences in polysaccharide synthesis between the mutated strains of RWY-1 and other strains, we measured the EPS content of the RWY-0, RWY-1, WY-2 and RWY-8 strains, and the enzyme activities of PGM and PGI, with the results shown in Fig 7.

As seen from Fig 7(A), the EPS content of the *G*. *lingzhi* mutant RWY-1 is higher than that of the control RWY-0. From Fig 7(B) and 7(C), we can see that the activities of the PGM and PGI enzymes in RWY-1 and RWY-2 are significantly higher than that of the control RWY-0. PGM and PGI are the key enzymes in the synthesis of *G*. *lingzhi* polysaccharides [20, 26, 27]. It has been reported that some genetically engineered *Ganoderma* strains can give rise to enhanced PGM and PGI activities [26, 27, 62]. In our case, we confirmed that the RWY-1 mutant strain had considerably improved polysaccharide production enzyme activities when compared with the WT RWY-0 strain.

### 3.4 Morphological assessment based on SEM observation

**Fig 8:**
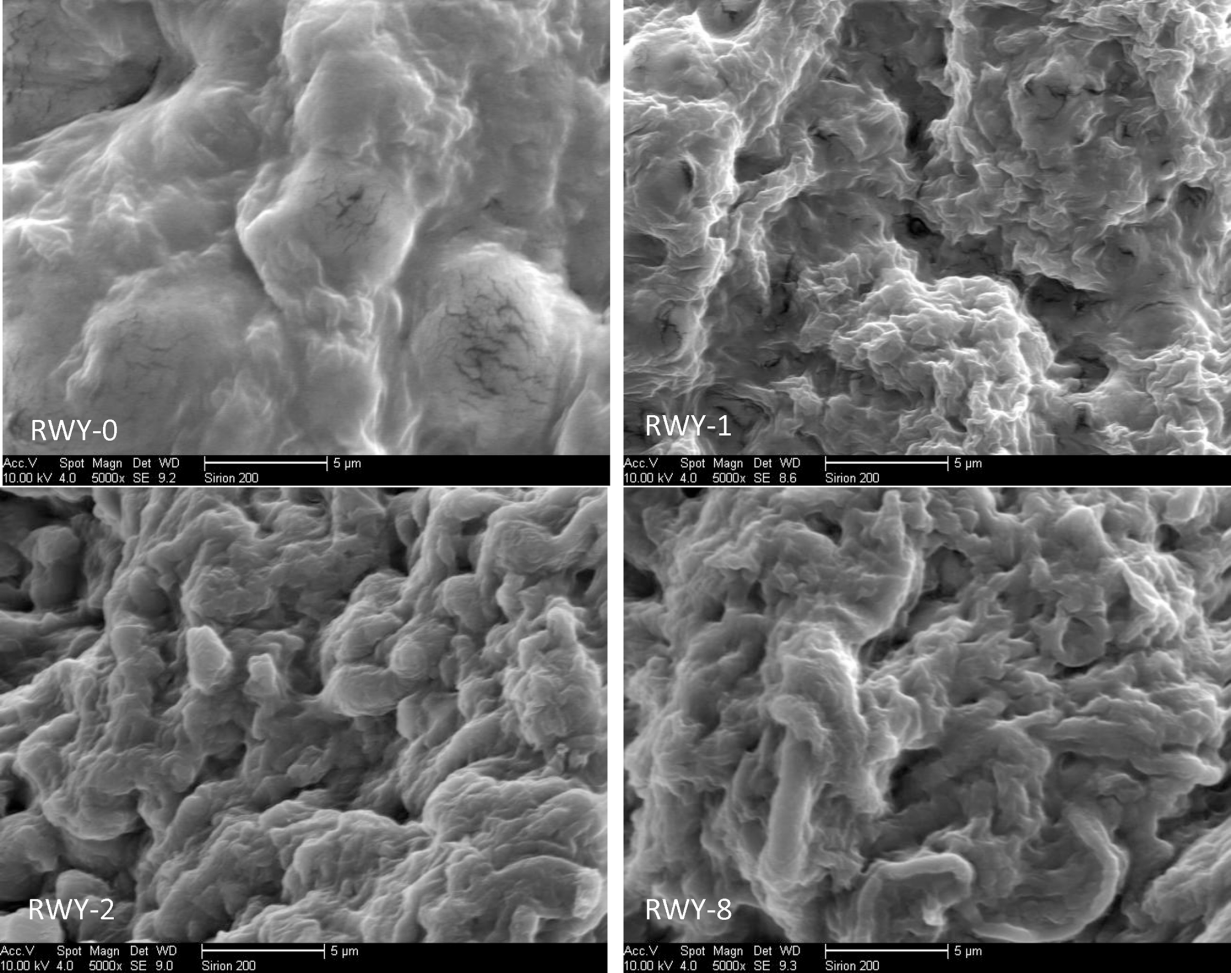
The scanning electron microscope (SEM) images of *G*. *lingzhi* mycelia (magnification 5000 times).

For the assessment of morphological changes of the mutant strains, the scanning electron microscope (SEM) images of the hyphae of different mutated strains were also recorded and compared, and the results are shown in Fig 8. It can be seen that there is big difference between the WT strain RWY-0 and the mutated strains, while there are certain small difference in mycelium morphology among the different mutated groups. More hyphae in the mutated mycelia resulted in a more concave-convex structure, more crack and sticks, while the surface of the control group was relatively flat. Concomitantly with the higher production of polysaccharides in RWY-1, the concave-convex structure is also the most prominent in RWY-1.

## 4. Conclusions

In summary, at least 10 mutated strains of *G*. *lingzhi* have been obtained by plasma mutagenesis. These mutagenized strains demonstrate significant differences in both the mycelium growth status and the *Ganoderma* polysaccharide content. The highest yield of polysaccharide production is found in RWY-1, with a 25.6% improvement in polysaccharide production. With the application of infrared spectroscopy (including both mid- and NIR-spectroscopy) in the mutant inspection, both qualitative and quantitative assessments of polysaccharides in mycelia of the mutagenized strains have been achieved. Therefore, this work demonstrates that a combination of plasma mutagenesis and infrared spectroscopy is very useful for *G*. *lingzhi* mutation breeding and screening research and application.

## Supporting information

S1 Fig Other evidence for the mutated strains: electrophoresis photographs of *G. lingzhi* strains treated with DBD plasma. Other evidence for the mutated strains: electrophoresis photographs of G. lingzhi strains treated with DBD plasma. WT: original strain; positive strain: other different strains, such as CGMCC 5.0026, G054, etc. all the mutated strains were comfirmed at least using 2 different primers. (a)~(d) RAPD identification for mutated strains, each agrose plate contained 12 strains of DNA amplified by two primers, D20 and D18. If one strain had different amplified product with both primers, the mutant was comfirmed. Note that (d) had indentified with no mutated strain. (e 1–2) showed the identification of 19 strains, with two primers, D20 and C5 of 12 primers was performed on each gel plate for 2 isolates.
(PDF)

S2 Fig The first derivative of NIR spectra of mutated *Ganoderma* mycelia. (PDF)

S3 Fig An NIR-based quantitative model for *Ganoderma* calibration and mutated strains. (PDF)

S1 Table Assignments of the characteristic mid-IR bands in the mid-IR spectrum of mutated *G. lingzhi* mycelium. (PDF)

S2 Table The predicted polysaccharide contents of *G. lingzhi* mutants. (PDF)

S3 Table Measurement of the polysaccharide content of mutated *G. lingzhi* strains based on the anthrone-sulfuric acid method. (PDF)

## References

[1] Marek S, Piotr R, Przemysław N, Anna B, Monika G, Kalač P, et al Comparison of multielemental composition of Polish and Chinese mushrooms (*Ganoderma* spp.). European Food Research and Technology. 2017;243(9):1555–1566.

[2] Upton R. American Herbal Pharmacopeia and Therapeutic Compendium: Reishi Mushroom, *Ganoderma* lucidum Standards of Analysis, Quality Control, and Therapeutics USA Canada: Santa Cruz 2000:1–28.

[3] United States Pharmacopoeia 39-National Formulary 34. United States Pharmacopoeia: Ganoderma Lucidum Fruiting Body Powder. 39 ed: Rockville, MD, USA,; 2016.

[4] Lv X, Chen D, Yang L, Zhu N, Li J, Zhao J, et al Comparative studies on the immunoregulatory effects of three polysaccharides using high content imaging system. International Journal of Biological Macromolecules. 2016;86(1):28–42. 26783639 10.1016/j.ijbiomac.2016.01.048

[5] Xiang QD, Yu Q, Wang H, Zhao MM, Liu SY, Nie S, et al Immunomodulatory activity of *Ganoderma* atrum polysaccharide on purified T lymphocytes through Ca2+/CaN and MAPK pathway based on RNA-seq. Journal of Agricultural & Food Chemistry. 2017;65(26):5306–5315. 28608696 10.1021/acs.jafc.7b01763

[6] Wu Q, Zhang H, Wang PG, Chen M. Evaluation of the efficacy and safety of *Ganoderma* lucidum mycelium-fermented liquid on gut microbiota and its impact on cardiovascular risk factors in human. Rsc Advances. 2017;7(71):45093–45100.

[7] Zhang YS, Li WJ, Zhang XY, Yan YX, Nie SP, Gong DM, et al *Ganoderma* atrum polysaccharide ameliorates anoxia/reoxygenation-mediated oxidative stress and apoptosis in human umbilical vein endothelial cells. International Journal of Biological Macromolecules. 2017;98:398–406. 10.1016/j.ijbiomac.2017.01.071 28108410

[8] Xiao C, Wu Q, Zhang J, Xie Y, Cai W, Tan J. Antidiabetic activity of *Ganoderma* lucidum polysaccharides F31 down-regulated hepatic glucose regulatory enzymes in diabetic mice. Journal of Ethnopharmacology. 2017;196:47–57. 10.1016/j.jep.2016.11.044 27902927

[9] Sargowo D, Wihastuti TA, Sukotjo CT, Anjani PM, Handayani O, Adrian LH. The Effect of Polysaccharides Peptides *Ganoderma* Lucidum to Aortic Foam Cell Count and Lipid Profile in Type 2 Diabetic Model Rattus Norvegicus Strain Wistar. 2017;9(3):9–153.

[10] Zhao Y, Feng C, Changlong FU, Hua H. Effect of Broken *Ganoderma* Lucidum Spore Powder on Serum ALT,AST Levels and Liver Inflammation in Mice with ConA-induced Immune Injury. Zhejiang Journal of Integrated Traditional Chinese & Western Medicine. 2017:760–764.

[11] Chen Y, Chen Q. Anti-Inflammatory and Hepatoprotective Effects of *Ganoderma* lucidum Polysaccharides on Carbon Tetrachloride-Induced Acute Liver Injury in Mice. Food Science. 2017:112–120.

[12] Sha C, Shan F. Improvement Function of *Ganoderma* Lucidum Polysaccharides on T Cell Subsets and AQP1, AQP3 Expression. Genomics & Applied Biology. 2018:16–22.

[13] Zeng P, Guo Z, Zeng X, Hao C, Zhang Y, Zhang M, et al Chemical, biochemical, preclinical and clinical studies of *Ganoderma* lucidum polysaccharide as an approved drug for treating myopathy and other diseases in China. Journal of cellular and molecular medicine. 2018:1–20. 10.1111/jcmm.13613 PMC6010762 29691994

[14] de Ana F, Luces S, Blanco R. PP-30 Clinical utility and safety of *Ganoderma* lucidum extract in acute lymphoblastic leukaemia as a adjuvant therapy: a case report. Archives of Disease in Childhood. 2017;102(10):27.

[15] Wang J, Zhang L, Yu Y, Cheung PC. Enhancement of antitumor activities in sulfated and carboxymethylated polysaccharides of *Ganoderma* lucidum. Journal of Agricultural & Food Chemistry. 2009;57(22):10565–10572. 19863048 10.1021/jf902597w

[16] Wang CL, Lu CY, Hsueh YC, Liu WH, Chen CJ. Activation of antitumor immune responses by *Ganoderma* formosanum polysaccharides in tumor-bearing mice. Applied Microbiology & Biotechnology. 2014;98(22):9389–9398. 25176445 10.1007/s00253-014-6027-6

[17] Zhao W, Xu JW, Zhong JJ. Enhanced production of ganoderic acids in static liquid culture of *Ganoderma* lucidum under nitrogen-limiting conditions. Bioresource Technology. 2011;102(17):8185 10.1016/j.biortech.2011.06.043 21742489

[18] Ren A, Qin L, Shi L, Dong X, Mu DS, Li YX, et al Methyl jasmonate induces ganoderic acid biosynthesis in the basidiomycetous fungus *Ganoderma* lucidum. Bioresource Technology. 2010;101(17):6785–6790. 10.1016/j.biortech.2010.03.118 20395130

[19] Papinutti L. Effects of nutrients, pH and water potential on exopolysaccharides production by a fungal strain belonging to *Ganoderma* lucidum complex. Bioresource Technology. 2010;101(6):1941–1946. 10.1016/j.biortech.2009.09.076 19846292

[20] Wei ZH, Liu L, Guo XF, Li YJ, Hou BC, Fan QL, et al Sucrose fed-batch strategy enhanced biomass, polysaccharide, and ganoderic acids production in fermentation of *Ganoderma* lucidum 5.26. Bioprocess & Biosystems Engineering. 2016;39(1):37–44. 26531749 10.1007/s00449-015-1480-x

[21] Tang YJ, Zhong JJ. Fed-batch fermentation of *Ganoderma* lucidum for hyperproduction of polysaccharide and ganoderic acid. Enzyme & Microbial Technology. 2002;31(1–2):20–28.

[22] Tang YJ, Zhong JJ. Role of oxygen supply in submerged fermentation of *Ganoderma* lucidum for production of *Ganoderma* polysaccharide and ganoderic acid. Enzyme & Microbial Technology. 2003;32(3–4):478–484.

[23] Tang YJ, Zhang W, Zhong JJ. Performance analyses of a pH-shift and DOT-shift integrated fed-batch fermentation process for the production of ganoderic acid and *Ganoderma* polysaccharides by medicinal mushroom *Ganoderma* lucidum. Bioresource Technology. 2009;100(5):1852–1859. 10.1016/j.biortech.2008.10.005 19010665

[24] Zhu LW, Zhong JJ, Tang YJ. Significance of fungal elicitors on the production of ganoderic acid and *Ganoderma* polysaccharides by the submerged culture of medicinal mushroom *Ganoderma* lucidum. Process Biochem. 2008;43(12):1359–1370.

[25] Huan-Jun L, De-Huai Z, Tong-Hui Y, Lu-Xi J, Xuya Y, Peng Z, et al Improved polysaccharide production in a submerged culture of *Ganoderma* lucidum by the heterologous expression of Vitreoscilla hemoglobin gene. Journal of Biotechnology. 2016;217:132–137. 10.1016/j.jbiotec.2015.11.011 26603122

[26] Xu JW, Ji SL, Li HJ, Zhou JS, Duan YQ, Dang LZ, et al Increased polysaccharide production and biosynthetic gene expressions in a submerged culture of *Ganoderma* lucidum by the overexpression of the homologous α-phosphoglucomutase gene. Bioprocess & Biosystems Engineering. 2015;38(2):399–405. 25218329 10.1007/s00449-014-1279-1

[27] Ji SL, Liu R, Ren MF, Li HJ, Xu JW. Enhanced Production of Polysaccharide Through the Overexpression of Homologous Uridine Diphosphate Glucose Pyrophosphorylase Gene in a Submerged Culture of Lingzhi or Reishi Medicinal Mushroom, *Ganoderma* lucidum (Higher Basidiomycetes). International Journal of Medicinal Mushrooms. 2015;17(5):435–442. 26082982 10.1615/intjmedmushrooms.v17.i5.30

[28] Twardowski T, Malyska A. Uninformed and disinformed society and the GMO market. Trends in Biotechnology. 2015;33(1):1–3. 10.1016/j.tibtech.2014.11.006 25528967

[29] Yolmeh M, Khomeiri M. Effect of mutagenesis treatment on antimicrobial and antioxidant activities of pigments extracted from Rhodotorula glutinis. Biocatalysis & Agricultural Biotechnology. 2017;10:285–290.

[30] Lackmann JW, Bandow JE. Inactivation of microbes and macromolecules by atmospheric-pressure plasma jets. Applied Microbiology & Biotechnology. 2014;98(14):6205–6213. 24841116 10.1007/s00253-014-5781-9

[31] Zengliang YU, Wang J, Yuan CL, Huang Q, Feng HY, Gong GH, et al Ion-beam-mutation breeding of an arachidonic acid biosynthesis microorganism and its industrial fermentation control. Chinese Science Bulletin. 2012:883–890.

[32] Peng R, Fu YZ, Zou J, Qiu H, Gan LT, Yi HL, et al Improvement of polysaccharide and triterpenoid production of *Ganoderma* lucidum through mutagenesis of protoplasts. Biotechnol Biotechnol Equip. 2016;30(2):381–387. 10.1080/13102818.2015.1133254 PubMed PMID: WOS:000372200700025.

[33] Ottenheim C, Nawrath M, Wu JC. Microbial mutagenesis by atmospheric and room-temperature plasma (ARTP): the latest development. Bioresources and Bioprocessing. 2018;5(1):1–14.

[34] Liu J, Chen J, Chen Z, Qin S, Huang Q. Isolation and characterization of astaxanthin-hyperproducing mutants of Haematococcus pluvialis (Chlorophyceae) produced by dielectric barrier discharge plasma. Phycologia. 2016;55(6):650–658.

[35] Li G, Yang F, Li R, Xu Z, Li B. A study on the breeding of new *Ganoderma* varieties by UV induced mutagenesis. Acta Microbiologica Sinica. 2001;41(2):229 12549031

[36] Ikehata H, Ono T. The mechanisms of UV mutagenesis. Journal of Radiation Research. 2011;52(2):115 21436607 10.1269/jrr.10175

[37] Hamelin C, Chung YS. Optimal conditions for mutagenesis by ozone in Escherichia coli K12. Mutation Research. 1974;24(3):271–279. 4606874 10.1016/0027-5107(74)90175-4

[38] L'Hérault P, Chung YS. Mutagenicity of ozone in different repair-deficient strains of Escherichia coli. Mol Gen Genet. 1984;197(3):472–477. 6396493 10.1007/BF00329945

[39] Xue Z, Zhang X, Wang L, Chong Z, Tan Y, Chang H, et al Recent progress on atmospheric and room temperature plasma mutation breeding technology and its applications. Ciesc Journal. 2014.

[40] Zhao B, Li Y, Li C, Yang H, Wang W. Enhancement of Schizochytrium DHA synthesis by plasma mutagenesis aided with malonic acid and zeocin screening. Applied Microbiology & Biotechnology. 2018;102(5):2351–2361. 29356868 10.1007/s00253-018-8756-4

[41] Liu X, Lv J, Xu J, Xia J, Dai B, Xu X, et al Erythritol production by Yarrowia lipolytica mutant strain M53 generated through atmospheric and room temperature plasma mutagenesis. Food Science & Biotechnology. 2017;26(4):1–8. 10.1007/s10068-017-0116-0 PMC6049542 30263627

[42] Li X, Zheng K, Lai C, Ouyang J, Yong Q. Improved Itaconic Acid Production from Undetoxified Enzymatic Hydrolysate of Steam-Exploded Corn Stover using an Aspergillus terreus Mutant Generated by Atmospheric and Room Temperature Plasma. BioResources. 2016;11(4):9047–9058.

[43] Cao S, Zhou X, Jin W, Wang F, Tu R, Han S, et al Improving of lipid productivity of the oleaginous microalgae Chlorella pyrenoidosa via atmospheric and room temperature plasma (ARTP). Bioresource Technology. 2017;244:1400–1406. 10.1016/j.biortech.2017.05.039 28539241

[44] Ma Y, He H, Wu J, Wang C, Chao K, Huang Q. Assessment of Polysaccharides from Mycelia of genus *Ganoderma* by Mid-Infrared and Near-Infrared Spectroscopy. Scientific Reports. 2018;8(1):1–10. 10.1038/s41598-017-17765-5 29311571 PMC5758644

[45] Wang JF, Jing LI, ITDatti Zhang J, Lin ZK, Lin ZX. Study on Classification of 21 Strains of *Ganoderma* by Antagonistic Effect,ITS and RAPD Technology. Southwest China Journal of Agricultural Sciences. 2017:26–33.

[46] Dwivedi S, Singh S, Chauhan UK, Tiwari MK. Inter and intraspecific genetic diversity (RAPD) among three most frequent species of macrofungi (*Ganoderma* lucidum, Leucoagricus sp. and Lentinus sp.) of Tropical forest of Central India. Journal of Genetic Engineering & Biotechnology. 2017. 10.1016/j.jgeb.2017.11.008 PMC6296601 30647715

[47] Barakat MN, Fattah RSA, Badr M, Eltorky MG. In vitro mutagenesis and identification of new variants via RAPD markers for improving Chrysanthemum morifolium. African Journal of Agricultural Research. 2010;5(8):748–757.

[48] Barakat MN, El-Sammak H. In vitro Mutagenesis, Plant Regeneration and Characterization of Mutants Via RAPD Analysis in Baby's Breath'Gypsophila paniculata L.'. Australian Journal of Crop Science. 2011;5(2):214–222.

[49] Li WJ, Tang X, Shuai XX, Jiang CJ, Liu X, Wang LF, et al Mannose receptor mediates the immune response to *Ganoderma* atrum polysaccharides in macrophages. J Agric Food Chem. 2017;65(2):348–357. 10.1021/acs.jafc.6b04888 27931102

[50] Liu AD, Zheng KY, Miao QF, Ikejima T, Zhang J, Fei XF. Effect of Polysaccharides from Fruit Body of *Ganoderma* tsugae on Bidirectional Regulation of Proinflammatory Cytokine Production in THP-1 Cells. Chem Res Chin Univ. 2009;25(4):487–491. PubMed PMID: WOS:000269232100019.

[51] National Pharmacopoeia Committee. Pharmacopoeia of the People’s Republic of China. Part. 2015;1:188–189.

[52] Lowry SR. Automated Spectral Searching in Infrared, Raman and Near‐Infrared Spectroscopy: John Wiley & Sons, Ltd; 2006.

[53] Liu J, Huang Q. Screening of Astaxanthin-Hyperproducing Haematococcus pluvialis Using Fourier Transform Infrared (FT-IR) and Raman Microspectroscopy. Applied Spectroscopy. 2016;70. 10.1177/0003702816645605 27296305

[54] Cote G, Ahlgren J, Smith M. Some Structural Features of An Insoluble Alpha-D-Glucan from Leuconostoc Mesenteroides Mutant Strain R1510. 1999:656–660. 10455497 10.1038/sj.jim.2900678

[55] Galichet A, Sockalingum GD, Belarbi A, Manfait M. Monitoring modifications in yeast cell wall: FTIR study of a genetically transformed S. cerevisiae strain: Springer Netherlands; 1999.

[56] Norris KH, Butler WL. Techniques for obtaining absorption spectra on intact biological samples. Ire Trans Biomed Electron. 1961;8(3):153–157. 13729604 10.1109/tbmel.1961.4322890

[57] Li-Chan ECY, Ismail AA, Sedman J, Voort FRVD. Vibrational Spectroscopy of Food and Food Products. Handbook of Vibrational Spectroscopy. 2006:4326–4359.

[58] Melin AM, Perromat A, Deleris G. Sensitivity of Deinococcus radiodurans to γ-Irradiation: A Novel Approach by Fourier Transform Infrared Spectroscopy. Archives of Biochemistry & Biophysics. 2001;394(2):265–274. 11594741 10.1006/abbi.2001.2533

[59] Liu JH, Song L, Huang Q. Rapid screening astaxanthin-hyperproducing Haematococcus pluvialis mutants through near infrared spectroscopy. Letters in Applied Microbiology. 2016;62(2):185–191. 10.1111/lam.12531 26643570

[60] Shao Y, Xie C, Jiang L, Shi J, Zhu J, He Y. Discrimination of tomatoes bred by spaceflight mutagenesis using visible/near infrared spectroscopy and chemometrics. Spectrochimica Acta Part A Molecular & Biomolecular Spectroscopy. 2015;140:431–436. 10.1016/j.saa.2015.01.018 25637814

[61] Wang XZ, Tang YY, Zhang JC, Cui FG, Chi YC, Wang CT. Analying the Effect of Chemical Treatment on Oil,Protein and Sucrose Content of Peanut with NIRS. Journal of Peanut Science. 2009:5–8.

[62] Tang YJ, Zhong JJ. Exopolysaccharide biosynthesis and related enzyme activities of the medicinal fungus, *Ganoderma* lucidum, grown on lactose in a bioreactor. Biotechnology Letters. 2002;24(12):1023–1026.

