# Supplementary material for: Improved production of polysaccharides in Ganoderma lingzhi mycelia by plasma mutagenesis and rapid screening of mutated strains through infrared spectroscopy
Source: PLoS One. 2018 Sep 21;13(9):e0204266. doi: 10.1371/journal.pone.0204266 (PMC6150529; doi:10.1371/journal.pone.0204266)
Supplement: S1 Fig — Other evidence for the mutated strains: electrophoresis photographs of G. lingzhi strains treated with DBD plasma. WT: original strain; positive strain: other different strains, such as CGMCC 5.0026, G054, etc. all the mutated strains were comfirmed at least using 2 different primers. (a)~(d) RAPD identification for mutated strains, each agrose plate contained 12 strains of DNA amplified by two primers, D20 and D18. If one strain had different amplified product with both primers, the mutant was comfirmed. Note that (d) had indentified with no mutated strain. (e 1–2) showed the identification of 19 strains, with two primers, D20 and C5 of 12 primers was performed on each gel plate for 2 isolates. (PDF) [file pone.0204266.s001.pdf]

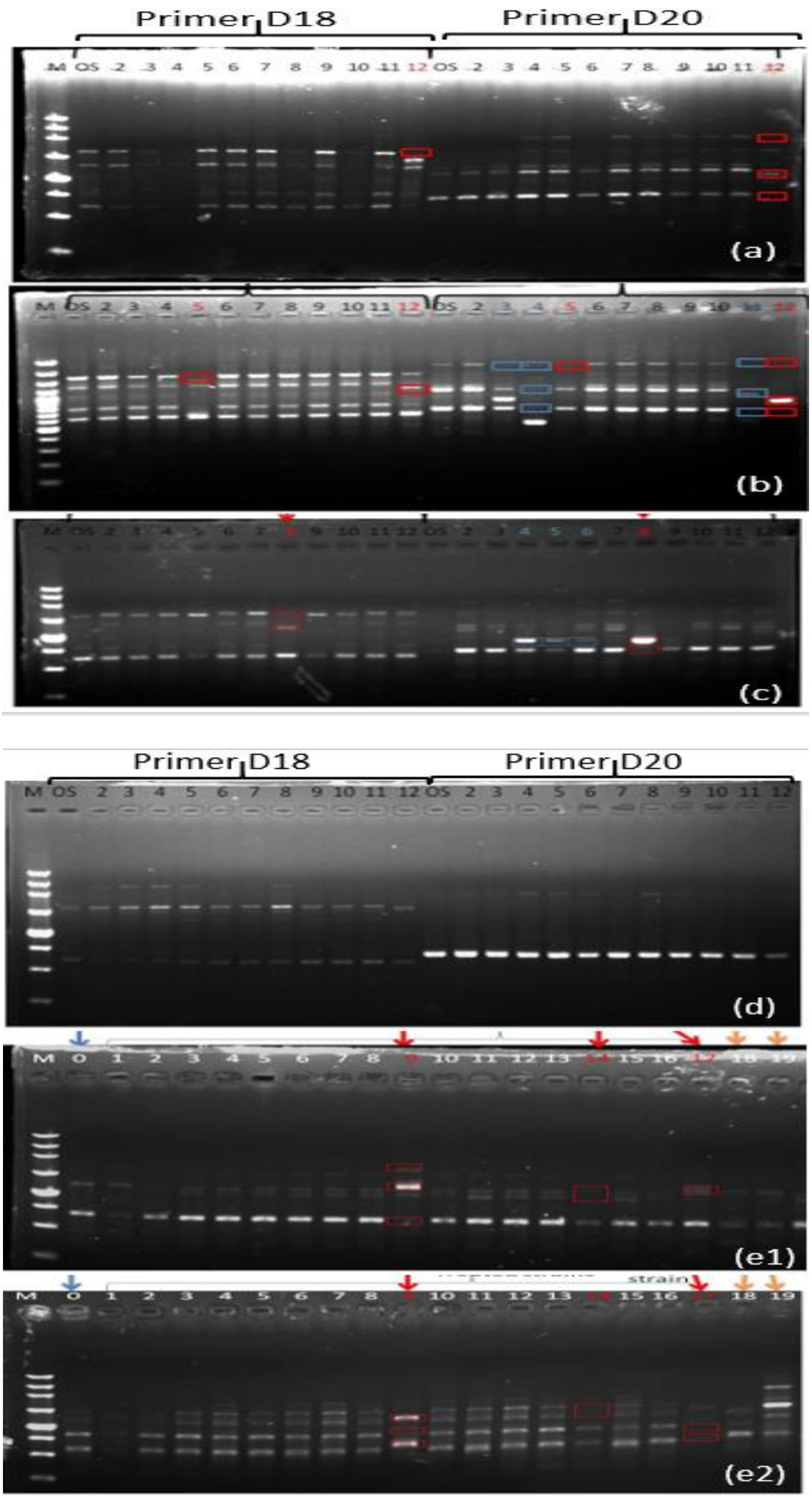

S1 Fig. Other evidence for the mutated strains: electrophoresis photographs of G. lingzhi strains treated with DBD plasma. WT: original strain; positive strain: other different strains, such as CGMCC 5.0026, G054, etc. all the mutated strains were comfirmed at least using 2 different primers. (a)~(d) RAPD identification for mutated strains, each agrose plate contained 12 strains of DNA amplified by two primers, D20 and D18. If one strain had different amplified product with both primers, the mutant was comfirmed. Note that (d) had indentified with no mutated strain. (e 1-2) showed the identification of 19 strains, with two primers, D20 and C5 of 12 primers was performed on each gel plate for 2 isolates.
