# Supplementary figures and images for: Improved production of polysaccharides in Ganoderma lingzhi mycelia by plasma mutagenesis and rapid screening of mutated strains through infrared spectroscopy
Source: PLoS One. 2018 Sep 21;13(9):e0204266. doi: 10.1371/journal.pone.0204266 (PMC6150529; doi:10.1371/journal.pone.0204266)

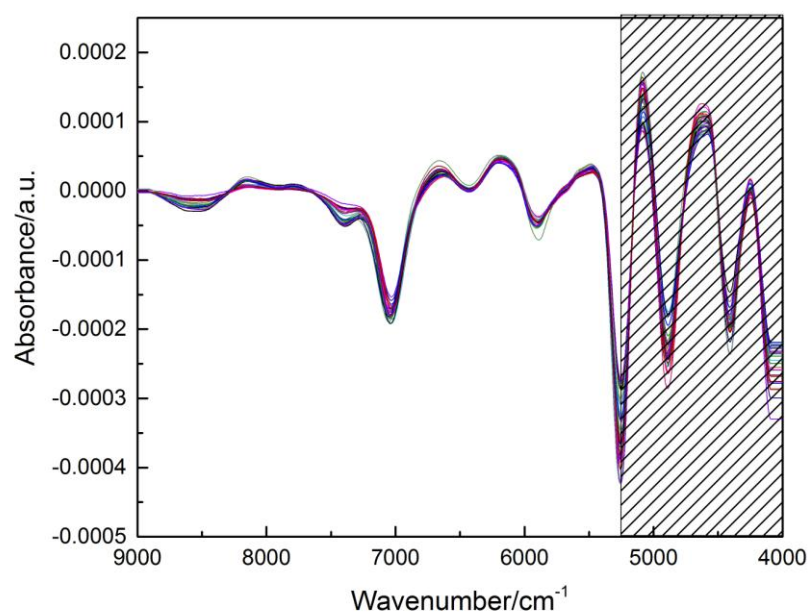

**S2 Fig.** The first derivative of NIR spectra of mutated *Ganoderma* mycelia

Supplement: S2 Fig — (PDF) [file pone.0204266.s002.pdf]

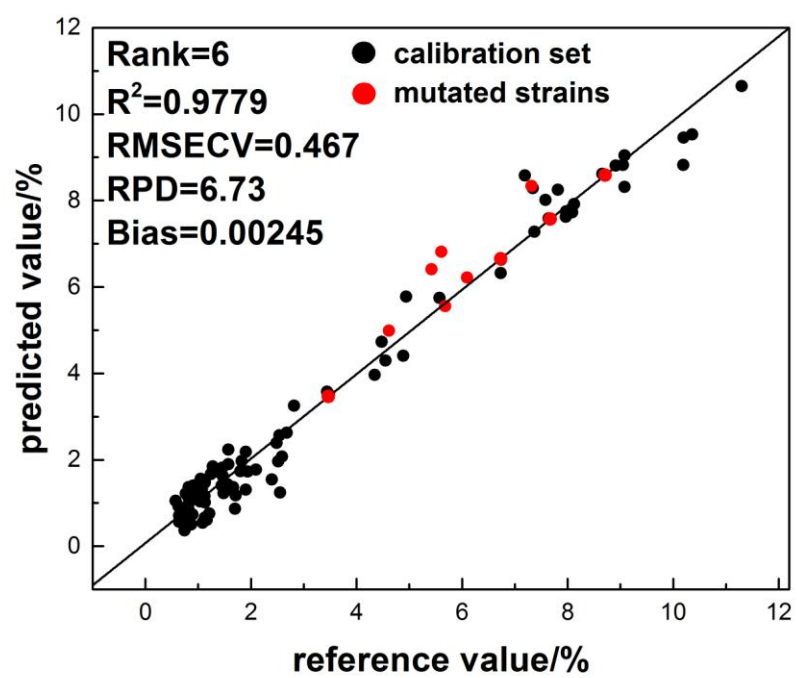

**S3 Fig.** NIR based quantitative model for *Ganoderma* calibration and mutated strains

Supplement: S3 Fig — (PDF) [file pone.0204266.s003.pdf]
