# Supplementary material for: Improved production of polysaccharides in Ganoderma lingzhi mycelia by plasma mutagenesis and rapid screening of mutated strains through infrared spectroscopy
Source: PLoS One. 2018 Sep 21;13(9):e0204266. doi: 10.1371/journal.pone.0204266 (PMC6150529; doi:10.1371/journal.pone.0204266)
Supplement: S1 Table — (PDF) [file pone.0204266.s004.pdf]

S1 Table Assignments of the characteristic mid-IR bands in the mid-IR spectrum of mutated *G. lingzhi* mycelium

Wavenumber(cm^{-1})	Functional Group Assignments
3400	-OH stretching
2925	CH_2 asymmetric stretching
1644	Amide Ⅰ
1450	CH2 in polysaccharides
1550	Amide Ⅱ
1425	C-H deformation in lignin and carbohydrates
1370	C-H in-plane bending vibration
1245	COH in-plane bending/CH in-plane bending
1153	C-O-C asymmetric stretching of glycosidic linkage
1078	C-O stretching of β-glucans
1044	stretching vibration of C-O-C group
1025	stretching vibration of C-O α-glycosidic bond
890	CH out-plane bending
