# Supplementary material for: Improved production of polysaccharides in Ganoderma lingzhi mycelia by plasma mutagenesis and rapid screening of mutated strains through infrared spectroscopy
Source: PLoS One. 2018 Sep 21;13(9):e0204266. doi: 10.1371/journal.pone.0204266 (PMC6150529; doi:10.1371/journal.pone.0204266)
Supplement: S2 Table — (PDF) [file pone.0204266.s005.pdf]

S2 Table The predicted polysaccharides contents of *G. lingzhi* mutant

Mutated stains	Polysaccharides contents predicted value/%
RWY-0	8.341±0.17
RWY-1	9.441±0.281*
RWY-2	7.796±0.277*
RWY-3	7.392±0.052
RWY-4	7.166±0.111
RWY-5	7.024±0.037
RWY-6	8.754±0.083
RWY-7	6.554±0.268*
RWY-8	4.791±0.333*
RWY-9	6.858±0.01
RWY-10	5.49±0.273*
