# Supplementary material for: Improved production of polysaccharides in Ganoderma lingzhi mycelia by plasma mutagenesis and rapid screening of mutated strains through infrared spectroscopy
Source: PLoS One. 2018 Sep 21;13(9):e0204266. doi: 10.1371/journal.pone.0204266 (PMC6150529; doi:10.1371/journal.pone.0204266)
Supplement: S3 Table — (PDF) [file pone.0204266.s006.pdf]

S3 Table Measurement of polysaccharide content of mutated *G. lingzhi* strains based on the anthrone-sulfuric acid method

Strain	polysaccharide content (%)
original srain	6.191±0.091
RWY-1	7.777±0.555
RWY-2	6.965±0.102
RWY-3	6.263±1.197
RWY-4	5.951±0.346
RWY-5	5.872±0.532
RWY-6	6.311±0.305
RWY-7	5.336±0.178
RWY-8	3.865±0.556
RWY-9	5.629±1.338
RWY-10	4.959±0.550
